# Association Between Income Inequality and County-Level COVID-19 Cases and Deaths in the US

**DOI:** 10.1001/jamanetworkopen.2021.8799

**Published:** 2021-05-03

**Authors:** Annabel X. Tan, Jessica A. Hinman, Hoda S. Abdel Magid, Lorene M. Nelson, Michelle C. Odden

**Affiliations:** 1 Department of Epidemiology and Population Health, Stanford University, Stanford, California

## Abstract

**Importance:**

Socioeconomically marginalized communities have been disproportionately affected by the COVID-19 pandemic. Income inequality may be a risk factor for SARS-CoV-2 infection and death from COVID-19.

**Objective:**

To evaluate the association between county-level income inequality and COVID-19 cases and deaths from March 2020 through February 2021 in bimonthly time epochs.

**Design, Setting, and Participants:**

This ecological cohort study used longitudinal data on county-level COVID-19 cases and deaths from March 1, 2020, through February 28, 2021, in 3220 counties from all 50 states, Puerto Rico, and the District of Columbia.

**Main Outcomes and Measures:**

County-level daily COVID-19 case and death data from March 1, 2020, through February 28, 2021, were extracted from the COVID-19 Data Repository by the Center for Systems Science and Engineering at Johns Hopkins University in Baltimore, Maryland.

**Exposure:**

The Gini coefficient, a measure of unequal income distribution (presented as a value between 0 and 1, where 0 represents a perfectly equal geographical region where all income is equally shared and 1 represents a perfectly unequal society where all income is earned by 1 individual), and other county-level data were obtained primarily from the 2014 to 2018 American Community Survey 5-year estimates. Covariates included median proportions of poverty, age, race/ethnicity, crowding given by occupancy per room, urbanicity and rurality, educational level, number of physicians per 100 000 individuals, state, and mask use at the county level.

**Results:**

As of February 28, 2021, on average, each county recorded a median of 8891 cases of COVID-19 per 100 000 individuals (interquartile range, 6935-10 666 cases per 100 000 individuals) and 156 deaths per 100 000 individuals (interquartile range, 94-228 deaths per 100 000 individuals). The median county-level Gini coefficient was 0.44 (interquartile range, 0.42-0.47). There was a positive correlation between Gini coefficients and county-level COVID-19 cases (Spearman ρ = 0.052; *P* < .001) and deaths (Spearman ρ = 0.134; *P* < .001) during the study period. This association varied over time; each 0.05-unit increase in Gini coefficient was associated with an adjusted relative risk of COVID-19 deaths: 1.25 (95% CI, 1.17-1.33) in March and April 2020, 1.20 (95% CI, 1.13-1.28) in May and June 2020, 1.46 (95% CI, 1.37-1.55) in July and August 2020, 1.04 (95% CI, 0.98-1.10) in September and October 2020, 0.76 (95% CI, 0.72-0.81) in November and December 2020, and 1.02 (95% CI, 0.96-1.07) in January and February 2021 (*P* < .001 for interaction). The adjusted association of the Gini coefficient with COVID-19 cases also reached a peak in July and August 2020 (relative risk, 1.28 [95% CI, 1.22-1.33]).

**Conclusions and Relevance:**

This study suggests that income inequality within US counties was associated with more cases and deaths due to COVID-19 in the summer months of 2020. The COVID-19 pandemic has highlighted the vast disparities that exist in health outcomes owing to income inequality in the US. Targeted interventions should be focused on areas of income inequality to both flatten the curve and lessen the burden of inequality.

## Key Points

**Question:**

How does the association between county-level income inequality, measured by the Gini coefficient, and COVID-19 cases and deaths change over time?

**Findings:**

This ecological cohort study found that there was a positive correlation between Gini coefficients and county-level COVID-19 cases and deaths during the study period. The association between income inequality and COVID-19 cases and deaths varied over time and was strongest in the summer months of 2020.

**Meaning:**

The findings suggest that, during the COVID-19 pandemic, areas of higher income inequality may serve as effective targets for interventions to mitigate the spread of SARS-CoV-2.

## Teaser

This cohort study evaluates the association between county-level income inequality and COVID-19 cases and deaths from March 2020 through February 2021 in bimonthly time epochs.

## Introduction

COVID-19, caused by SARS-CoV-2, has resulted in the largest pandemic in a century. The United States has been impacted significantly, accounting for 25% of COVID-19 cases and deaths from COVID-19 worldwide.^1^ A significant body of evidence has shown that the prevalence of cases and deaths due to COVID-19 has been disproportionately higher among socially marginalized communities, exacerbated by health disparities.^2,3,4,5,6,7,8^ One recent study by Liao and De Maio^9^ found that an increase in a county’s income inequality corresponded with an increase in COVID-19 incidence. Another study by Oronce et al^10^ reported an association between increased state-level income inequality and COVID-19 cases. Income inequality may increase opportunities for infection, as the most disadvantaged individuals need to stay in the labor force to afford to live in a region that also includes much wealthier residents.^11^ Moreover, individuals with lower incomes are more likely to reside in crowded housing and have public-facing jobs, such as service, child and elder care, and cleaning or janitorial services, which can increase the risk of exposure to SARS-CoV-2.^12^

In this study, we sought to evaluate the correlation between county-level income inequality, measured by the Gini coefficient, and county-level COVID-19 case and death counts across the United States at different time epochs in 2020 and 2021. County-level income inequality reflects the lived experience of those residing in these regions better than state-level measures. Moreover, many public health orders are implemented at the county level, making this geographical unit relevant for policy. We hypothesized that counties with worse income inequality would have higher numbers of COVID-19 cases and deaths compared with those with more income equality and that the association between income inequality and COVID-19 cases would have strengthened over time, as those who reside in communities with more income equality would have a greater ability to implement risk mitigation strategies.

## Methods

In this ecological cohort study, the number of cases and deaths from COVID-19 were extracted from March 1, 2020, to February 28, 2021, from the COVID-19 Data Repository by the Center for Systems Science and Engineering at Johns Hopkins University in Baltimore, Maryland. County-level data were obtained from the 2014 to 2018 American Community Survey 5-year estimates.^13^ Health system data (physicians per 100 000 individuals) were obtained from Area Health Resources Files.^14^ Self-reported data on mask use were obtained from *New York Times* estimates.^15^ Our primary explanatory variable of interest was the Gini coefficient, presented as a value between 0 and 1, where 0 represents a perfectly equal geographical region where all income is equally shared and 1 represents a perfectly unequal society where all income is earned by 1 individual.^16^ Potential confounders at the county level were obtained from the 2014 to 2018 American Community Survey estimates, and included the following: poverty, age, race/ethnicity, crowding (given by occupancy per room), urbanicity and rurality, educational levels, number of physicians per 100 000 individuals, and mask use. We also included state as a fixed effect. To examine the time interaction between cases and deaths and Gini coefficients, noncumulative cases and deaths were split into time epochs spanning 2 months each beginning with the World Health Organization declaration of the pandemic: March and April 2020, May and June 2020, July and August 2020, September and October 2020, November and December 2020, and January and February 2021. We selected 2-month epochs to evaluate temporal trends in the course of the pandemic in the US. We have chosen to use a bimonthly time scale to account for seasonality and because this is a large enough time unit to accrue sufficient case and death counts in counties in which these numbers were small. We propose that March and April 2020 represent early pandemic months, May and June 2020 represent late spring, July and August 2020 represent summer, September and October 2020 represent fall and back to school season, November and December 2020 represent winter and the US holiday season, and January and February 2021 represent winter and postholiday travel. The Stanford University institutional review board determined that this study did not require institutional review board review because all data are deidentified and available publicly. This study followed the Strengthening the Reporting of Observational Studies in Epidemiology (STROBE) reporting guideline.^17^

### Statistical Analysis

We explored the associations between county-level Gini coefficients and county-level COVID-19 cases and deaths. Gini coefficients were transformed by dividing by 0.05 for easier interpretation in the models.^18^ We used negative binomial regression to account for overdispersion in unadjusted and adjusted analyses. We used a likelihood ratio test to evaluate the interaction between county-level Gini coefficients and county-level COVID-19 cases and deaths. Confounders in the adjusted analyses included median percentage of population living at or below the federal poverty level, median percentage of population by age (aged <25 years, 25-39 years, 40-64 years, 65-79 years, 80-84 years, and ≥85 years), median percentage of population by race (White, Black, Asian, Native American, Hawaiian, and Pacific Islander), median percentage of population of Hispanic ethnicity, median percentage of crowding (given by occupancy per room: ≤0.50, 0.51-1.00, 1.01-1.50, 1.51-2.00, and ≥2.01), median percentage of house ownership and rental, median percentage of population living in an urban or rural area, median percentage of population by educational level (less than high school, high school, some college, and college), and time epochs. As a proxy for the accessibility of health care, we included number of physicians per 100 000 individuals as a confounder. To account for county-level disease control policies, we adjusted for self-reported mask use (never, sometimes, frequently, and always). To assess for median poverty rate as an effect modifier, we also examined the association between COVID-19 cases and deaths and the interaction between income inequality and COVID-19 stratified by time epochs. All *P* values were from 2-sided tests, and results were deemed statistically significant at *P* < .05.

## Results

As of February 28, 2021, there were a total of 28 306 349 cases of COVID-19 and 505 620 deaths from COVID-19 across 3220 counties across 50 states, Puerto Rico, and the District of Columbia. On average, each county recorded a median of 8891 cases per 100 000 individuals (interquartile range, 6935-10 666 cases per 100 000 individuals) and 156 deaths per 100 000 individuals (interquartile range, 94-228 deaths per 100 000 individuals) (Table 1). The median county-level Gini coefficient was 0.44 (interquartile range, 0.42-0.47). We mapped county-level distributions of both Gini coefficients and total number of cases per 100 000 individuals, which shows a weak positive correlation between the 2 during the study period (Spearman ρ = 0.052; *P* < .001; Figure). Similarly, there was a weak positive correlation between Gini coefficients and total deaths per 100 000 individuals (Spearman ρ = 0.134; *P* < .001). The association of inequality and COVID-19 cases and deaths varied over time. Each 0.05-unit higher Gini coefficient (greater inequality) was associated with an adjusted relative risk of COVID-19 cases: 1.18 (95% CI, 1.13-1.24) in March and April 2020, 1.23 (95% CI, 1.18-1.29) in May and June 2020, 1.28 (95% CI, 1.22-1.33) in July and August 2020, 0.90 (95% CI, 0.87-0.94) in September and October 2020, 0.85 (95% CI, 0.81-0.88) in November and December 2020, and 1.02 (95% CI, 0.98-1.07) in January and February 2021 (*P* < .001 for interaction) (Table 2). Similarly, for deaths, for each 0.05-unit higher Gini coefficient, the adjusted relative risk of COVID-19 deaths was 1.25 (95% CI, 1.17-1.33) in March and April 2020, 1.20 (95% CI, 1.13-1.28) in May and June 2020, 1.46 (95% CI, 1.37-1.55) in July and August 2020, 1.04 (95% CI, 0.98-1.10) in September and October 2020, 0.76 (95% CI, 0.72-0.81) in November and December 2020, and 1.02 (95% CI, 0.96-1.07) in January and February 2021 (*P* < .001 for interaction).

**Table 1.** Description of County Characteristics

Variable of interest	Median per 100 000 (IQR) (N = 3220)
Cases, No. (overall)	8891 (6935-10 666)
March and April 2020	59 (23-143)
May and June 2020	176 (66-426)
July and August 2020	655 (337-1246)
September and October 2020	1094 (602-1746)
November and December 2020	3573 (2466-4803)
January and February 2021	2245 (1480-2909)
Deaths, No. (overall)	156 (94-228)
March and April 2020	0 (0-5)
May and June 2020	1 (0-11)
July and August 2020	7 (0-21)
September and October 2020	13 (3-30)
November and December 2020	43 (21-78)
January and February 2021	46 (24-76)

Abbreviation: IQR, interquartile range.

**Figure.**
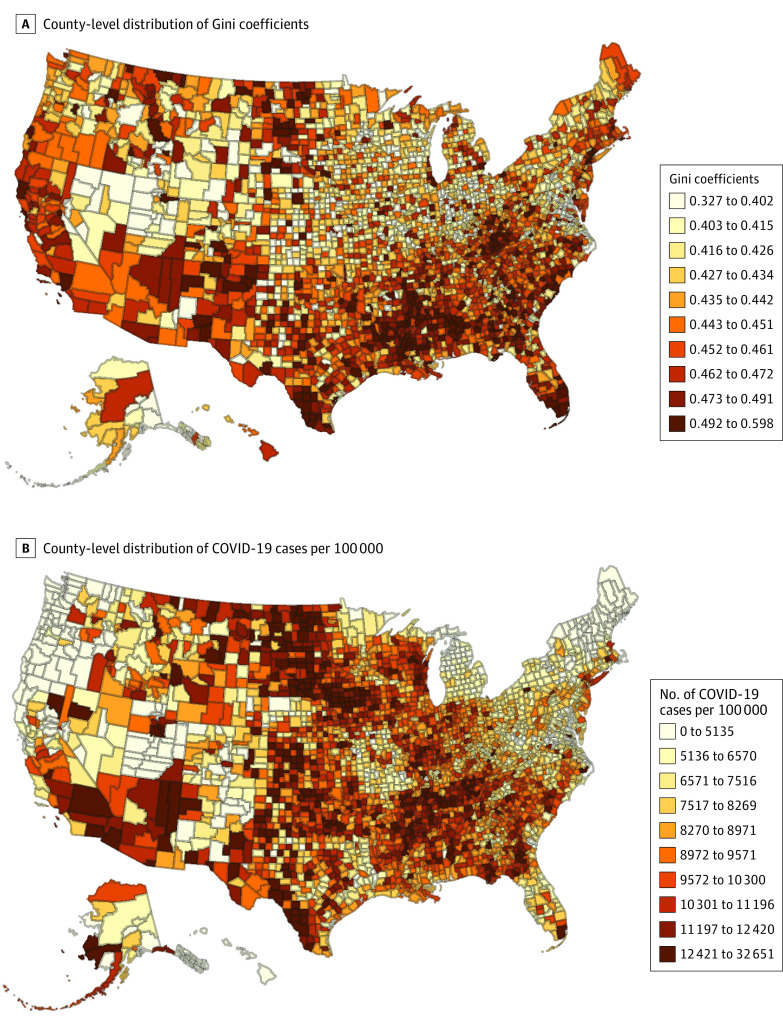
County-Level Distribution of Percentile of Gini Coefficients and COVID-19 Cases From March 1, 2020, to February 28, 2021

**Table 2.** Association Between Gini Coefficients and COVID-19 Cases and Deaths by Time Epoch at the County Level

Characteristic	Relative risk (95% CI)						*P* value^a^
	March and April 2020	May and June 2020	July and August 2020	September and October 2020	November and December 2020	January and February 2021	
Cases, No.^b^	323	465	1007	932	3244	2554	
Unadjusted	1.31 (1.25-1.36)	1.28 (1.23-1.34)	1.38 (1.33-1.44)	0.94 (0.90-0.98)	0.89 (0.85-0.92)	1.07 (1.03-1.12)	<.001
Adjusted	1.18 (1.13-1.24)	1.23 (1.18-1.29)	1.28 (1.22-1.33)	0.90 (0.87-0.94)	0.85 (0.81-0.88)	1.02 (0.98-1.07)	<.001
Deaths, No.^b^	18.1	20.2	16.7	14.1	35.2	48.0	
Unadjusted	1.54 (1.44-1.64)	1.47 (1.38-1.56)	1.70 (1.59-1.81)	1.18 (1.11-1.26)	0.83 (0.79-0.88)	1.11 (1.05-1.18)	<.001
Adjusted	1.25 (1.17-1.33)	1.20 (1.13-1.28)	1.46 (1.37-1.55)	1.04 (0.98-1.10)	0.76 (0.72-0.81)	1.02 (0.96-1.07)	<.001

^a^ For difference across epoch.

^b^ Per 100 000 people in the US.

To account for any differences observed in the association between income inequality and COVID-19 burden that might be modified by high or low median poverty rate, we examined the interaction between income inequality and COVID-19. We also looked at the interaction when stratified by time epochs. We found no significant interactions.

## Discussion

Our findings build on emerging evidence that economic disparities are positively associated with the risk of SARS-COV-2 infection and COVID-19 deaths. Our results show that counties with greater levels of income inequality frequently have higher numbers of COVID-19 cases and deaths. This association varied over time, strengthening over the summer and peaking in July and August 2020. However, the association was inverse for cases in September through December 2020 and deaths in November and December 2020.

Our findings are consistent with the recent work of Liao and De Maio,^9^ who reported that a 1.0% increase in a county’s income inequality was associated with an adjusted risk ratio of 1.02 for COVID-19 cases, although this study did not examine differences over time. Another study by Oronce et al^10^ found that there was a positive unadjusted correlation between state-level income inequality measured by the Gini coefficient and the number of cases and deaths due to COVID-19 and that the association with COVID-19 deaths was independent of potential confounders. Our findings build on this work by evaluating variability in the strength of the association between income inequality and COVID-19 cases and deaths at the county level over time.^12^ Moreover, our work accounts for additional potential confounding factors, such as crowding and urban or rural living, as well as measures of deprivation (poverty, housing situation, educational level, and health system presence).

We hypothesize that a potential mechanism explaining the association between COVID-19 cases and Gini coefficients being strongest in the summer months is that individuals with lower incomes in counties with greater income inequality may be at higher risk for COVID-19 infection owing to the economic pressure to remain in high-risk employment. Many who are at increased risk of COVID-19 cannot work from home.^19^ Individuals with lower incomes tend to work in sectors that produce nontradable goods such as restaurants, hotels, or entertainment venues, which require person-to-person contact.^20^ However, because this was an ecological study, we cannot make any inferences at the individual level.

We observed a change in the direction of the association between income inequality and COVID-19 cases in September through December 2020 and deaths in November and December 2020. In these epochs, higher income inequality was associated with a lower rate of cases and deaths. We hypothesize that there may have been increased social mixing in these fall months, likely owing to a combination of factors including a policy shift from the White House away from risk mitigation strategies, increased individual risk-taking behavior (ie, “pandemic fatigue”), a return to in-person schooling and college education, and the Thanksgiving and Christmas holiday season with increased travel both within states and out of state. It is possible that the direct association of income inequality with COVID-19 cases and death was nullified by these factors, which led to an increase in cases and death. However, this hypothesis remains speculative, and future studies using GPS (Global Positioning System) patterns during this era may better elucidate social distancing behavior stratified by income inequality.

### Limitations

This study has some limitations, including that it is an ecological design with a county-level outcome measure; as such, individual risk cannot be extrapolated from it. Furthermore, we did not account for concurrent changes in measures of income and employment owing to the time-lag availability of these measures in the American Community Survey. In addition, we did not pursue another form of analysis, such as a time-series analysis, because of the interest in evaluating month-to-month changes and because some counties had small numbers of cases and death. Last, the association between COVID-19 risk and income inequality may be stronger than estimated owing to the fact that some of the confounders included, such as crowded housing, may likely also be mediators on the pathway, which resulted in an attenuated risk.

## Conclusions

The COVID-19 pandemic has highlighted the vast inequality across all socioeconomic levels in the United States. The findings of this cohort study suggest an association between county-level income inequality and COVID-19 cases and deaths. Targeted interventions implemented in a timely manner are of vital importance, especially as the United States turns a new corner with COVID-19 control and vaccine rollout. Targeted interventions should be focused on areas of income inequality to both flatten the curve and lessen the burden of inequality. Potential targeted interventions include the distribution of personal protective equipment, enhanced COVID-19 testing, providing further guidance on COVID-19 nonpharmaceutical interventions, educational campaigns, and finally, improving vaccine acceptance among those at highest risk of exposure.

## References

[1] Dong E, Du H, Gardner L. An interactive web-based dashboard to track COVID-19 in real time. Lancet Infect Dis. 2020;20(5):533-534. doi:10.1016/S1473-3099(20)30120-1 32087114 PMC7159018

[2] Bello-Chavolla OY, González-Díaz A, Antonio-Villa NE, . Unequal impact of structural health determinants and comorbidity on COVID-19 severity and lethality in older Mexican adults: considerations beyond chronological aging. J Gerontol A Biol Sci Med Sci. 2021;76(3):e52-e59. doi:10.1093/gerona/glaa163 32598450 PMC7337730

[3] Hawkins D. Social determinants of COVID-19 in Massachusetts, United States: an ecological study. J Prev Med Public Health. 2020;53(4):220-227. doi:10.3961/jpmph.20.256 32752590 PMC7411251

[4] Kim SJ, Bostwick W. Social vulnerability and racial inequality in COVID-19 deaths in Chicago. Health Educ Behav. 2020;47(4):509-513. doi:10.1177/1090198120929677 32436405 PMC8183499

[5] Millett GA, Jones AT, Benkeser D, . Assessing differential impacts of COVID-19 on Black communities. Ann Epidemiol. 2020;47:37-44. doi:10.1016/j.annepidem.2020.05.003 32419766 PMC7224670

[6] Tai DBG, Shah A, Doubeni CA, Sia IG, Wieland ML. The disproportionate impact of COVID-19 on racial and ethnic minorities in the United States. Clin Infect Dis. 2021;72(4):703-706. doi:10.1093/cid/ciaa815 32562416 PMC7337626

[7] Yancy CW. COVID-19 and African Americans. JAMA. 2020;323(19):1891-1892. doi:10.1001/jama.2020.6548 32293639

[8] Krieger N, Waterman PD, Chen JT. COVID-19 and overall mortality inequities in the surge in death rates by zip code characteristics: Massachusetts, January 1 to May 19, 2020. Am J Public Health. 2020;110(12):1850-1852. doi:10.2105/AJPH.2020.305913 33058698 PMC7662002

[9] Liao TF, De Maio F. Association of social and economic inequality with coronavirus disease 2019 incidence and mortality across US counties. JAMA Netw Open. 2021;4(1):e2034578. doi:10.1001/jamanetworkopen.2020.34578 33471120 PMC7818127

[10] Oronce CIA, Scannell CA, Kawachi I, Tsugawa Y. Association between state-level income inequality and COVID-19 cases and mortality in the USA. J Gen Intern Med. 2020;35(9):2791-2793. doi:10.1007/s11606-020-05971-3 32583336 PMC7313247

[11] Chetty R, Stepner M, Abraham S, . The association between income and life expectancy in the United States, 2001-2014. JAMA. 2016;315(16):1750-1766. doi:10.1001/jama.2016.4226 27063997 PMC4866586

[12] Pan W, Miyazaki Y, Tsumura H, Miyazaki E, Yang W. Identification of county-level health factors associated with COVID-19 mortality in the United States. J Biomed Res. 2020;34(6):437-445. doi:10.7555/JBR.34.20200129 33109778 PMC7718079

[13] United States Census Bureau. American Community Survey (ACS) 5-year estimate releases for 2014–2018. Accessed August 18, 2020. https://api.census.gov/data/2018/acs/acs5/subject/variables.html

[14] United States Health Resources & Services Administration. Area Health Resources Files. Published 2020. Updated July 31, 2020. Accessed February 8, 2021. https://data.hrsa.gov/topics/health-workforce/ahrf

[15] Katz J, Sanger-Katz M, Quealy K. A detailed map of who is wearing masks in the U.S. *New York Times*. July 17, 2020. Accessed January 29, 2021. https://www.nytimes.com/interactive/2020/07/17/upshot/coronavirus-face-mask-map.html

[16] De Maio FG. Income inequality measures. J Epidemiol Community Health. 2007;61(10):849-852. doi:10.1136/jech.2006.052969 17873219 PMC2652960

[17] von Elm E, Altman DG, Egger M, Pocock SJ, Gøtzsche PC, Vandenbroucke JP; STROBE Initiative. The Strengthening the Reporting of Observational Studies in Epidemiology (STROBE) statement: guidelines for reporting observational studies. Ann Intern Med. 2007;147(8):573-577. doi:10.7326/0003-4819-147-8-200710160-00010 17938396

[18] Yitzhaki S, Schechtman E. The Gini Methodology: A Primer on a Statistical Methodology. Springer; 2012. doi:10.1007/978-1-4614-4720-7

[19] Baker MG. Nonrelocatable occupations at increased risk during pandemics: United States, 2018. Am J Public Health. 2020;110(8):1126-1132. doi:10.2105/AJPH.2020.305738 32552016 PMC7349441

[20] Eichenbaum MS, Rebelo S, Trabandt M. Inequality in life and death. December 31, 2020. Accessed February 28, 2021. https://www.kellogg.northwestern.edu/faculty/rebelo/htm/inequality.pdf

